# Flightless-I governs cell fate by recruiting the SUMO isopeptidase SENP3 to distinct *HOX* genes

**DOI:** 10.1186/s13072-017-0122-8

**Published:** 2017-03-23

**Authors:** Arnab Nayak, Anja Reck, Christian Morsczeck, Stefan Müller

**Affiliations:** 1 Institute of Biochemistry II, Goethe University Medical School, University Hospital Building 75, Theodor-Stern-Kai 7, 60590 Frankfurt am Main, Germany; 2 Department of Oral and Maxillofacial Surgery, University of Regensburg, 93042 Regensburg, Germany

**Keywords:** Sumoylation, MLL1/2, *HOX* gene, Mesenchymal stem cells, Flightless-I, Chromatin, SILAC-based proteomics

## Abstract

**Background:**

Despite recent studies on the role of ubiquitin-related SUMO modifier in cell fate decisions, our understanding on precise molecular mechanisms of these processes is limited. Previously, we established that the SUMO isopeptidase SENP3 regulates chromatin assembly of the MLL1/2 histone methyltransferase complex at distinct *HOX* genes, including the osteogenic master regulator *DLX3*. A comprehensive mechanism that regulates SENP3 transcriptional function was not understood.

**Results:**

Here, we identified flightless-I homolog (FLII), a member of the gelsolin family of actin-remodeling proteins, as a novel regulator of SENP3. We demonstrate that FLII is associated with SENP3 and the MLL1/2 complex. We further show that FLII determines SENP3 recruitment and MLL1/2 complex assembly on the *DLX3* gene. Consequently, FLII is indispensible for H3K4 methylation and proper loading of active RNA polymerase II at this gene locus. Most importantly, FLII-mediated SENP3 regulation governs osteogenic differentiation of human mesenchymal stem cells.

**Conclusion:**

Altogether, these data reveal a crucial functional interconnection of FLII with the sumoylation machinery that converges on epigenetic regulation and cell fate determination.

**Electronic supplementary material:**

The online version of this article (doi:10.1186/s13072-017-0122-8) contains supplementary material, which is available to authorized users.

## Background

The ubiquitin-like SUMO (small ubiquitin-like modifier) system has emerged as a key regulator of cell function [1–5]. SUMO covalently modifies its target proteins and has important implications not only in adult life but also during early development [6, 7]. The multistep, ATP-dependent SUMO conjugation pathway requires an enzymatic (E1–E2–E3) cascade for the formation of an isopeptide bond between the carboxy-terminal di-glycine motif of SUMO paralogs (SUMO1 and the highly related SUMO2/3) and lysine residues in a target protein. The modification is reversed by specific cysteine proteases, commonly termed SUMO-specific isopeptidases or SUMO proteases. These enzymes cleave the isopeptide bond between the SUMO moiety and substrates. SUMO-specific isopeptidases belong to three distinct families, viz. the Ulp/SENP, the Desi and the USPL1 family [8–10]. In humans, six different SENP isoforms (SENP1, 2, 3, 5, 6 and 7) with distinct subcellular localizations, substrate specificities and functions have been identified. For example, SENP3 exerts preferential cleavage activity toward SUMO2-/3-modified substrates. It is distributed in the nucleolus and nucleoplasm and shuttles between these two compartments in an mTOR-controlled process [11]. Work from our group and others has initially established a role of SENP3 in ribosome maturation [11–14]. Subsequently, we and others could also unravel a crucial function of SENP3 in the control of gene expression [15–17], in particular in the regulation of homeobox (*HOX*) genes [18].

The coordinated expression of *HOX* genes that encode homeodomain-containing transcription factors is critical for embryonic development [19–21]. In adult tissues, *HOX* genes are involved in regulating cell commitment pathways such as hematopoietic differentiation [22]. The distal-less (Dll) family of *HOX* genes, which encodes DLX transcription factors, has a critical role in limb development, brain patterning and craniofacial morphogenesis [23]. *DLX3* exerts key functions in osteogenic differentiation, and accordingly loss of *DLX3* expression is a signature of defective osteogenic differentiation processes [24–27].

The expression of *HOX* genes is exquisitely regulated by the epigenetic modifiers, trithorax (*trxG*)- and Polycomb (*PcG*)-group proteins, which activate or repress *HOX* genes, respectively [28–32]. The mixed lineage leukemia (MLL)–histone methyltransferase (HMT) complex proteins belong to the trxG group and catalyze trimethylation of lysine 4 on histone 3 (H3K4me3) [33, 34]. This is typically associated with an active state of chromatin and thus found on transcriptionally active genes or developmentally regulated bivalent promoters [35–40]. In mammals, distinct subtypes of MLL complexes are found. They all consist of a SET1/MLL methyltransferase (SET1A/B, MLL1, 2, 3 or 4) as the catalytic core [41–43]. However, for optimum catalytic activity the core requires the association with a multiprotein module minimally composed of WDR5, RbBP5, Ash2L and DPY-30, known as WRAD module [44]. The ordered and timed assembly of the WRAD module at chromatin is one critical point in the control of SET1/MLL function [41, 45]. Our earlier work established a SUMO-dependent mechanism of this assembly process at a subset of *HOX* genes. We had shown that the MLL1/2 complex subunit RbBP5 is covalently modified by SUMO2. We further demonstrated that the SUMO isopeptidase SENP3 catalyzes the desumoylation of RbBP5, which is a prerequisite for the recruitment of Ash2L and menin into functional MLL1/2 complexes at a subset of *HOX* genes, including the *DLX3* gene [18]. However, how SENP3 itself is regulated and targeted to perform its transcription regulatory role remained unclear.

Here, we identify flightless-I homolog (FLII) as a central player in this process. FLII has initially been described as an actin-remodeling protein that belongs to the evolutionary conserved gelsolin protein superfamily [46–48]. The FLII protein consists of an N-terminal leucine-rich repeat (LRR) domain involved in protein–protein interaction. The C-terminus of FLII is made up of the gelsolin-like domain, which mediates actin binding and protein–protein interaction. FLII function has been assigned to cytoskeleton organization during cell migration and negative regulation of wound repair [49]. Apart from cytoskeleton-related functions, FLII has also been shown to act as a transcriptional co-activator in nuclear receptor signaling [50].

In this study, we identify FLII as a major regulator of SENP3. We demonstrate that FLII regulates the chromatin association of SENP3, thereby modulating MLL1/2 complex activity. Through this pathway, FLII governs *DLX3* gene expression and determines osteogenic differentiation process of human mesenchymal stem cells.

## Result

### FLII is a major interactor of SENP3

Our earlier work unraveled a functional and physical association of SENP3 with pre-60S ribosomes and MLL1/2 histone methyltransferase complexes [13, 14, 18]. A major interaction platform for SENP3 at pre-60S ribosomes is the PELP1 complex, but how SENP3 is targeted to chromatin and the MLL1/2 complex is currently unknown. In order to investigate these issues, we performed a system-wide interactome study for endogenous SENP3 using SILAC (stable isotope labeling in cell culture)-based mass spectrometry (MS). Cell lysates from HeLa cells labeled with light or heavy amino acids were incubated with either control or anti-SENP3 antibodies for immunoprecipitation. Following separation by SDS-PAGE, proteins were excised from gels, trypsinized and peptides measured by MS. After applying a number of stringent filtering criteria (as detailed in the legends to Additional file 1: Figs. S1 and S2), a cytoscape (version 3.3.0) map of the SENP3 interactome was generated. The map is comprised of 27 proteins that satisfied multilayered filtering criteria. Among the SENP3-associated proteins with the highest SILAC ratio, highest peptide count and lowest PEP score, we consistently found FLII (Additional file 1: Fig. S1, Additional file 2: Table S1).

When these 27 high-confident proteins were analyzed in STRING database [51], a high clustering coefficient of 0.863 was observed (Additional file 1: Fig. S2). This indicates that the proteins enriched by SENP3 IP are high probability clusters of functionally related protein groups. Accordingly, when the SENP3 cytoscape cluster was integrated in the known FLII network extracted from STRING database (version 10.0), we found another FLII interacting protein LRRFIP1 as a common high confidence candidate identified in the SENP3 interactome (Additional file 1: Fig. S2b). This further indicates that SENP3 is connected to FLII.

**Fig. 1:**
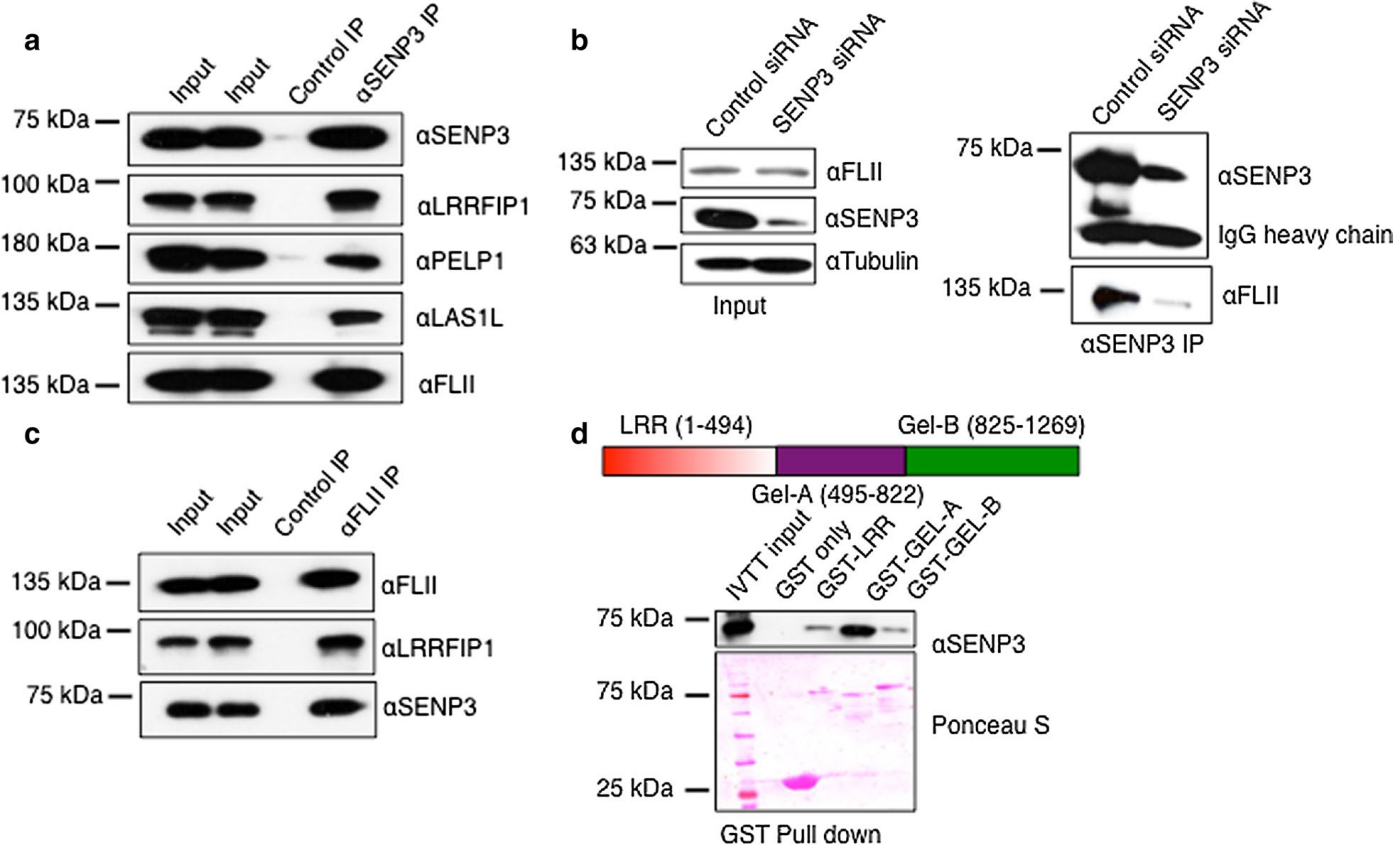
FLII is a major novel interactor of SENP3. **a** Immunoprecipitation using anti-SENP3 antibody was performed on extracts from HeLa cells. Immunoprecipitates were analyzed by SDS-PAGE and probed with antibodies as indicated. **b** Similar to **a** except, SENP3 was depleted from HeLa cells by siRNA. After 72 h of siRNA transfection, SENP3 IP was performed. SDS-PAGE and immunoblotting with selected antibodies were performed as indicated. **c** Same as **b** but antibody directed against FLII was used for immunoprecipitation. **d** Schematic representation of FLII domain structure. The *numbers in brackets* indicate the amino acids. Equal amounts of in vitro transcribed/translated SENP3 were mixed with either GST only as a control or with GST-tagged FLII fragments (GST-LRR, GST-GelA and GST-GelB) and GST pull-down was performed. After intensive washing, bound proteins were eluted in SDS-sample buffer, separated by SDS-PAGE and probed for the presence of SENP3

To validate the MS data, directed co-immunoprecipitation assays were performed. To this end, we first expressed a Flag-tagged SENP3 version in HeLa cells by transient transfection and performed anti-Flag IPs. In this setup, endogenous FLII was specifically captured on anti-Flag beads (Additional file 1: Fig. S3a). When immunoprecipitating endogenous SENP3, we detected the known SENP3 interactors PELP1 and LAS1L upon immunoblotting with the respective antibodies. Moreover, we strongly enriched for endogenous FLII and LRRFIP1 in the anti-SENP3 IP, but not in the IgG control IP (Fig. 1a). Notably, upon depletion of SENP3 by siRNA, FLII was lost in the anti-SENP3 IPs, ruling out that the SENP3–FLII co-IP results from an antibody cross-reaction (Fig. 1b). Moreover, in a reverse anti-FLII-IP, endogenous SENP3 as well as LRRFIP1 was detected by immunoblotting with the respective antibodies (Fig. 1c).

To probe for direct interaction between FLII and SENP3 and to map the binding domain of SENP3 in FLII, we performed GST pull-down assays using recombinantly expressed fragments of FLII. The LRR, GelA and GelB domains were fused to GST and used as baits in pull-down assays to test for binding to SENP3, which was generated by in vitro transcription/translation in a rabbit reticulocyte-based system (Fig. 1d). The experiment revealed a physical interaction of SENP3 predominantly with the GelA domain of FLII.

Previous reports have described both nuclear and cytosolic localizations of FLII [47, 48, 52]. To see in which compartment the SENP3–FLII interaction occurs, cellular proteins were separated in a nuclear and cytosolic fraction and IPs were performed. Anti-FLII immunoprecipitation from the different fractions indicates that the FLII–SENP3 interaction is mostly nuclear (Additional file 1: Fig. S3b). In accordance with this, immunofluorescence assays revealed the distribution of both proteins in the nucleoplasm (Additional file 1: Fig. S3c). Taken together, these observations establish a robust and specific nuclear interaction of SENP3 with FLII via its GelA domain.

### FLII is involved in the regulation of SENP3-controlled *DLX3* gene expression

**Fig. 2:**
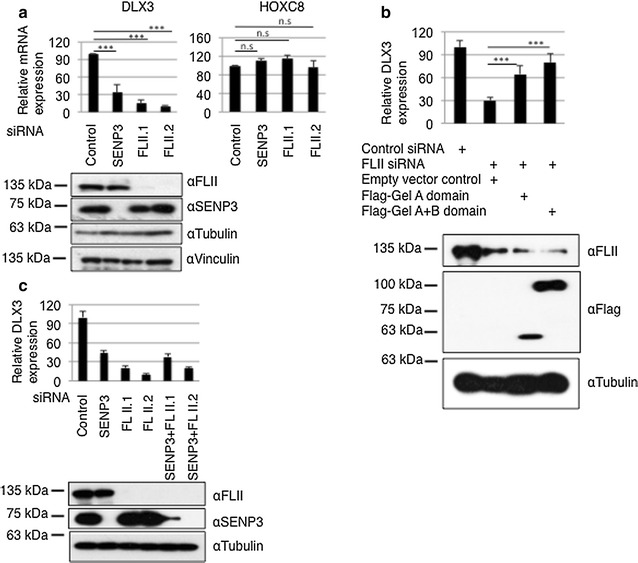
FLII controls *DLX3* gene expression. **a** FLII was depleted from HeLa by two independent siRNAs. Total RNA was prepared from control cells or cells transfected with either SENP3 or FLII siRNA. RNA was reverse transcribed and cDNA was used in real-time quantitative PCR (RT-qPCR) to monitor the expression of *DLX3* and *HOXC8*. Normalized values (against GAPDH mRNA level) represent the average of three independent experiments performed in triplicate ± SEM (*n* = 3, *T* test, ***p < 0.001, *n.s.* not significant). The *lower panel* demonstrates efficient depletion of SENP3 or FLII. **b** Same as in **a** except flag-FLII domains were expressed. Data represent the average values from three independent experiments in duplicate ± SEM. **c** Same as in **a** except co-depletion of FLII together with SENP3 was performed. The *lower panel* is a representative western blot, demonstrating efficient depletion of SENP3 and FLII

Next, we addressed the physiological relevance of FLII binding to SENP3. Because we had defined *DLX3* as a SENP3-regulated gene, *DLX3* expression was chosen as a model system [18]. We initially monitored *DLX3* mRNA levels by RT-qPCR in control cells or in cells depleted from FLII by two different siRNAs. Intriguingly, when compared to controls, depletion of FLII with either siRNA results in a fivefold–tenfold down-regulation of *DLX3* mRNA expression (Fig. 2a). Expression of the siRNA-resistant Gel A or GelA/B domains of FLII restores the *DLX3* gene expression to nearly 65 and 80%, respectively (Fig. 2b). Notably, FLII protein depletion had almost no effect on expression of the homeobox gene *HOXC8* (Fig. 2a), which is also not sensitive to SENP3 depletion [18]. To get a first clue whether FLII and SENP3 dictate DLX3 expression via a common pathway, we co-depleted both proteins by siRNA. Importantly, when compared to individual depletion of either SENP3 or FLII, co-depletion of both proteins did not further decrease DLX3 mRNA levels indeed supporting the idea that SENP3 and FLII act in a common pathway in controlling DLX3 expression (Fig. 2c).

**Fig. 3:**
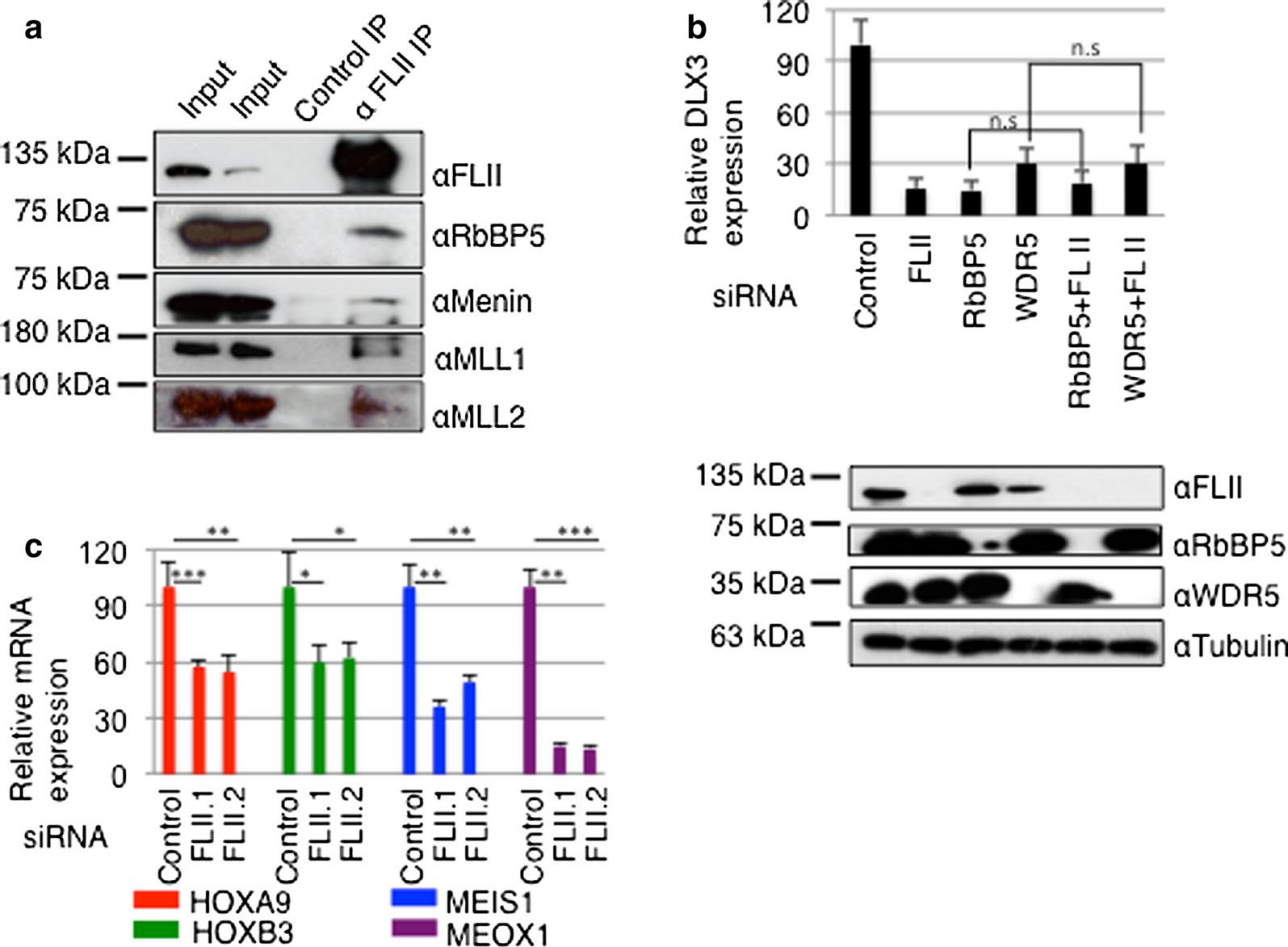
FLII is functionally associated with MLL1/2 complexes and governs *HOX* gene expression. **a** Endogenous FLII was immunoprecipitated from HeLa cell extracts, and the immunoprecipitates were probed for the presence of RbBP5, menin, MLL1 or MLL2 as indicated. **b** Same as Fig. 2a except total RNA was prepared from control cells or cells individually depleted for FLII, RbBP5 and WDR5 (SET1/MLL component subunit) by siRNAs or co-depleted for FLII and the indicated SET1/MLL components. RNA was reverse transcribed and cDNA was used in RT-qPCR to monitor *DLX3* gene expression. Data represent as the average of three independent experiments performed in triplicate ± SEM. **c** FLII was depleted from HeLa by two independent siRNAs, and RT-qPCR was performed to monitor the expression of *HOXA9*, *HOXB3*, *MEIS1* and *MEOX1* (*n* = 3, *T* test, *p < 0.05; **p < 0.01; ***p < 0.001, *n.s.* not significant)

In our previous work, we demonstrated that SENP3 is associated with MLL1/2 methyltransferase complexes and exerts control on DLX3 expression by regulating the assembly of these complexes. Based the above physical association of SENP3 and FLII, we therefore asked whether FLII is also associated with MLL1/2 components. To address this, we performed endogenous anti-FLII IPs and tested for the presence of components of MLL1/2 complexes in the immunoprecipitated material. We detected RbBP5, menin, WDR5 and the catalytic core subunits MLL1 and MLL2, but not the related Set1A protein in the FLII immunoprecipitate (Fig. 3a; Additional file 1: Fig. S4a–c). Moreover, by employing GST pull-down assays we observed a physical interaction between RbBP5 and FLII mediated mostly through the GelA domain (Additional file 1: Fig. S4d and e).

To reveal a functional connection of FLII to the MLL1/2 methyltransferase complex, we co-depleted cells from FLII together with distinct components of the MLL1/2 complexes. Similar to what was observed upon co-depleting SENP3 and FLII, co-depletion of RbBP5 or WDR5 together with FLII did not further reduce the level of DLX3 mRNA when compared to their single depletion (Fig. 3b). These observations support the idea of a common signaling pathway involving SENP3, FLII and MLL1/2 complexes in the execution of *DLX3* expression.

Next, we asked whether FLII regulates a broader spectrum of *HOX* genes. To this end, we investigated the effect of FLII depletion on the expression of a set of selected *HOX* genes—*HOXA9, HOXB3, MEIS1* and *MEOX1*—that were previously defined as SENP3-responsive genes [18]. Importantly, depletion of FLII by two different siRNAs resulted in down-regulation of mRNA transcripts of all four genes (Fig. 3c) and further strengthened the idea that FLII is involved in regulating the expression of *HOX* genes in conjunction with SENP3.

Noteworthy, the transcriptional function of FLII has previously connected to the estrogen receptorα (ERα) pathway (50). However, since ERα is not expressed in HeLa cells, the effects described here cannot be assigned to an interplay of FLII with ERα. Moreover, in the ERα-positive MCF7 cell line, SENP3 does not act in conjunction with FLII in ERα-mediated gene regulation (Additional file 1: Fig. S5a).

### SENP3 recruitment to its target gene *DLX3* is dependent on FLII

We next aimed to determine the molecular mechanism of FLII-controlled SENP3 function. As the catalytic activity of SENP3 is indispensible for *DLX3* gene expression, we first checked whether FLII has any role in modulating SENP3 catalytic activity. However, a SENP3 activity assay in HeLa cell lysates from control cells or cells depleted from FLII did not reveal differences in catalytic activity (Additional file 1: Fig. S5b and c).

**Fig. 4:**
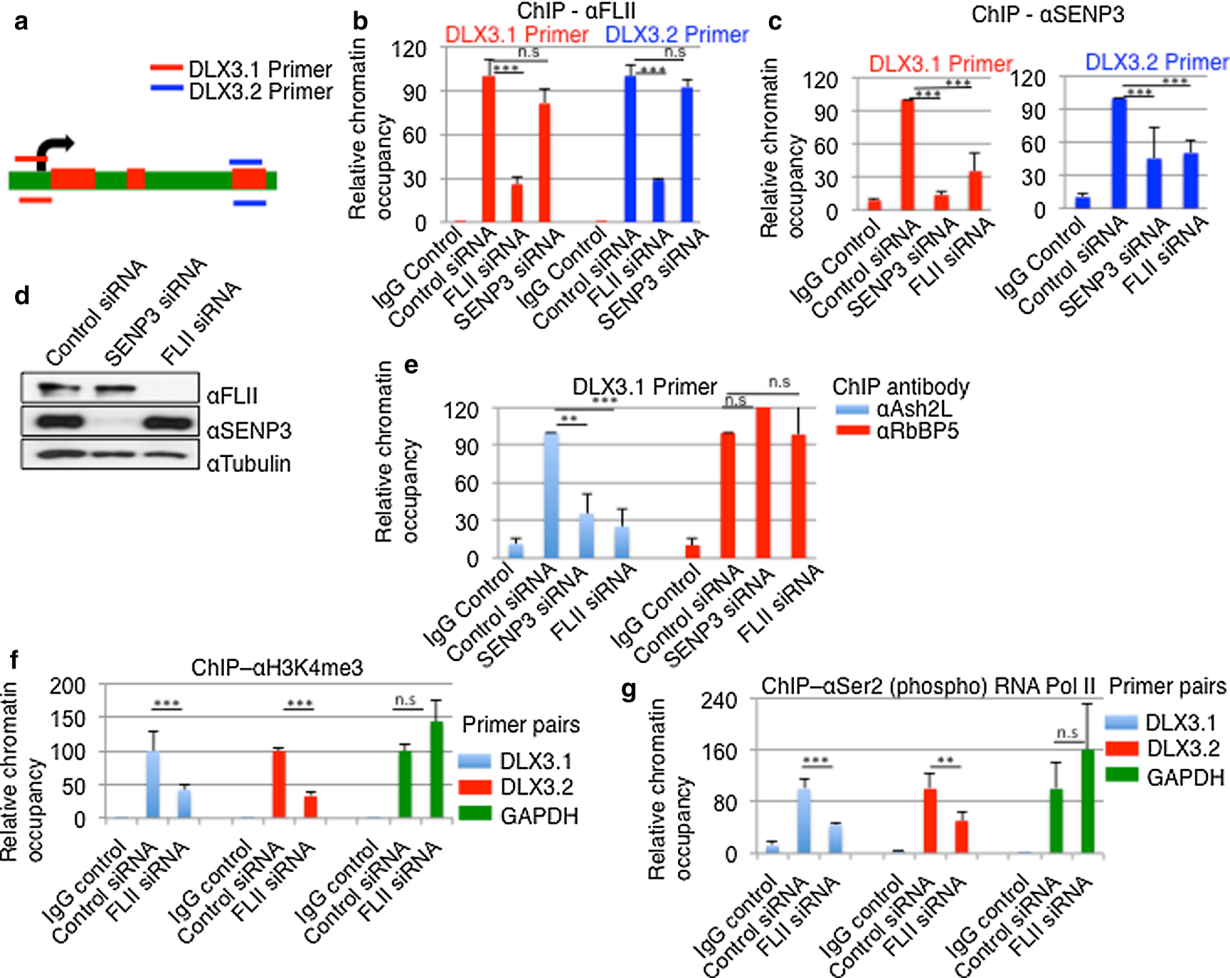
FLII controls SENP3 targeting to the *DLX3* gene and regulates MLL1/2 complex assembly. **a** Schematic representation of the human *DLX3* locus is shown with transcription start site marked as *arrow* and exons as *red boxes*. Primer pairs (DLX3.1 and DLX3.2) covering promoter or exon 3 region are indicated by *color lines*. **b** Chromatin was isolated from control HeLa cells or cells depleted from SENP3 and FLII, and ChIP assays were performed with anti-FLII antibodies. Primer pairs DLX3.1 and DLX3.2 were used to amplify DNA recovered from immunoprecipitated chromatin. Values are the average of three independent experiments performed in triplicate ± SEM (*T* test, **p < 0.01; ***p < 0.001, *n.s.* not significant). **c** Same as **b** except SENP3 antibody was used for ChIP. **d** HeLa cell lysate was probed with indicated antibodies to monitor the depletion of FLII and SENP3. **e** Same as **c**, except anti-Ash2L and anti-RbBP5 antibodies were used in ChIP. **f** Same as **c**, except anti-H3K4me3 antibody was used for ChIP and GAPDH primer was included in the qPCR as a control. **g** Same as **f**, except anti-ser2 (phospho) RNA Pol II antibody was used for ChIP

We next focused on a possible direct role of FLII at chromatin and therefore asked whether FLII is present at the *DLX3* gene. To address this issue, ChIP (chromatin immunoprecipitation) assays were performed in order to monitor the binding of SENP3 and FLII in the promoter region or exon 3 of *DLX3* gene as we had previously detected SENP3 at these sites (Fig. 4a) [18]. Cross-linked chromatin–protein complexes from HeLa cells indeed revealed a strong enrichment of exogenous Flag-tagged FLII as well as endogenous FLII on the *DLX3* gene locus (Additional file 1: Fig. S6a; Fig. 4b). Importantly, enrichment of FLII on chromatin was not influenced by depletion of SENP3 (Fig. 4b), indicating that FLII was recruited to chromatin independently from SENP3. These results led us to the idea that FLII may in turn target SENP3 to chromatin and in particular to the *DLX3* gene locus. To test this idea, ChIP assays with an anti-SENP3 antibody were performed as above, and immunopurified *DLX3* gene fragments were quantified by qPCR. Importantly, depletion of FLII leads to a more than threefold reduction of SENP3 at the *DLX3* gene locus, both in the promoter and in the exon regions (Fig. 4c). Noteworthy, this reduction was not due to an altered stability or expression of SENP3, as SENP3 levels remained unchanged upon FLII depletion (Fig. 4d; Additional file 1: Fig. S6b). As SENP3 is required for proper chromatin recruitment of Ash2L and menin [18], we wondered whether defective chromatin residency of SENP3 in the absence of FLII might also affect MLL1/2 complex assembly. Therefore, we followed the chromatin association of Ash2L on the *DLX3* gene by ChIP as described above. Importantly, FLII depletion perfectly phenocopied the loss of SENP3 with respect to Ash2L residency at the *DLX3* gene. Very similar to what was observed upon depletion of SENP3, Ash2L occupancy at the *DLX3* promoter is reduced to 20–30%. However, RbBP5, another core subunit of the MLL1/2 complexes, as well as MLL1 and MLL2 itself remains associated with the *DLX3* gene in the absence of either FLII or SENP3 (Fig. 4e; Additional file 1: Fig. S6c–e). Noteworthy, FLII depletion did not affect the protein level of any of the core components of MLL1/2 complexes, ruling out that the reduced chromatin occupancy in the absence of FLL is due to changes in protein stability (Additional file 1: Fig. S7a, b). Importantly, FLII depletion had no effect on Ash2L chromatin association at the *HOXC8* gene, which is consistent with the observation that expression of HOXC8 was not sensitive to either FLII or SENP3 depletion (Additional file 1: Fig. S7c). Altogether, this indicates that FLII is involved in proper SENP3 targeting to distinct *HOX* genes, thereby controlling the assembly and function of MLL1/2 complexes.

To further validate this idea, we monitored H3K4 trimethylation (H3K4me3) at the *DLX3* gene, because this epigenetic mark is catalyzed by MLL1/2 complexes. H3K4me3 can be assayed by ChIP experiments using an antibody directed against this modification. In FLII-depleted cells, H3K4me3 marks at the *DLX3* gene were reduced to 30–40%, when compared to control cells. It is worth noting that no reduction of H3K4 trimethylation was detected on the unrelated control gene *GAPDH* (Fig. 4f) or *HOXC8* (Additional file 1: Fig. S7d). H3K4me3 is typically considered as a chromatin mark for active genes, and accordingly active RNA Pol II is found on genes that harbor H3K4-trimethyl marks. In particular, the serine 2 phosphorylated form of RNA Pol II C-terminal domain (CTD) is a signature of productive transcription, and it overlaps with H3K4-trimethyl-enriched chromatin domains [53]. We therefore monitored the presence of ser2P-RNA Pol II at the *DLX3* gene locus in the presence or absence of FLII. Again, we observed more than twofold reduction of Pol II residency at the *DLX3* gene, but not on the control *GAPDH* or *HOXC8* gene, when endogenous FLII is knocked down (Fig. 4g; Additional file 1: Fig. S7e). Collectively, these data suggest that FLII governs chromatin association of SENP3 on specific *HOX* genes, thereby regulating MLL1/2 complex assembly, which ultimately impinges on gene activation.

To further strengthen the idea that the FLII recruits SENP3 to the MLL complex assembly on distinct gene loci at chromatin, we performed FLII siRNA followed by immunoprecipitation of endogenous SENP3 and probed for co-precipitation of RbBP5 and vice versa. When compared to control cells, RBBP5 is much reduced in the anti-SENP3 IP upon loss of FLII. Even more strikingly SENP3 does not co-immunoprecipitate with RbBP5 in the absence of FLII (Additional file 1: Fig. S7f). Importantly, however, SENP3 depletion did not affect Ash2L interaction with RbBP5 further supporting our notion that SENP3 does not generally affect the assembly of MLL complexes in solution, but acts at chromatin on distinct *HOX* genes. (Additional file 1: Fig. S7g).

### FLII regulates sumoylation of RbBP5 and is critical for differentiation of human dental follicle stem cells

**Fig. 5:**
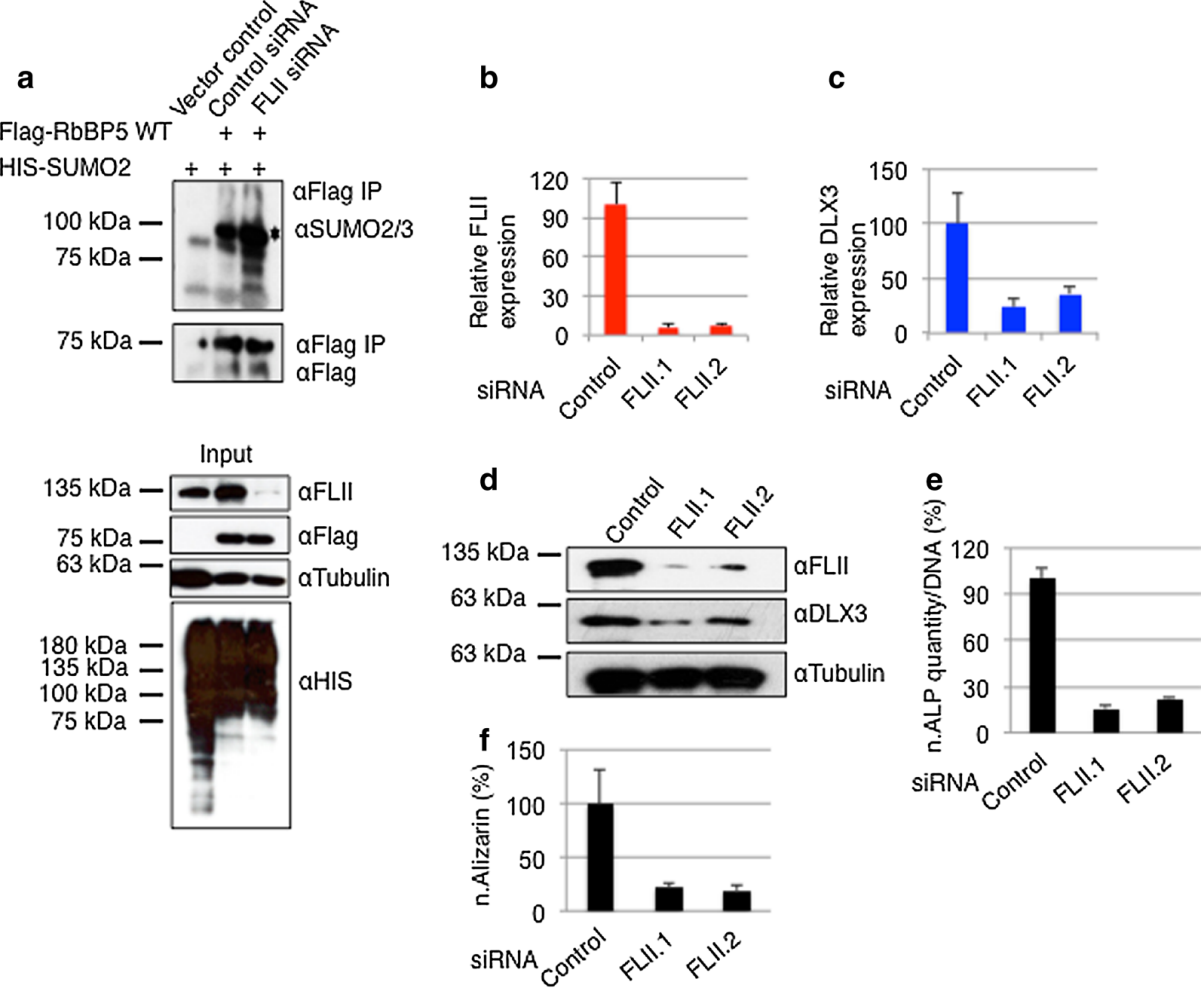
FLII regulates RbBP5 sumoylation and osteogenic differentiation of DFCs. **a** HeLa cells treated with control siRNA or FLII siRNA were transfected with Flag-RbBP5 and SUMO2 as indicated, and SUMOylation of RbBP5 was monitored by anti-SUMO2 immunoblotting following anti-Flag-IP. The *asterisk* represents the sumoylated RbBP5 species. **b**, **c** Total RNA was isolated from DFCs, after treating cells with two independent FLII siRNAs or control siRNA. Expression of FLII (**b**) and DLX3 mRNA (**c**) was determined by RT-qPCR. Values (normalized for GAPDH expression) represent the mean of three experiments ± SEM. **d** Cell lysates originating from DFCs (**b**, **c**) were separated by SDS-PAGE and analyzed by immunoblotting with the indicated antibodies. **e** ALP activity was quantified in differentiating DFCs, 10 days after transfection with FLII siRNAs or control siRNA and incubation with osteogenic differentiation medium (ODM). **f** Mineral deposits in DFC cultures were measured by alizarin red staining after 5 weeks of culture with ODM and transfection with two independent FLII siRNAs or a control siRNA

In our previous work, we had shown that the MLL1/2 complex subunit RbBP5 is covalently modified by SUMO2. We further demonstrated that SENP3 catalyzes the desumoylation of RbBP5, which is a prerequisite for the recruitment of Ash2L and menin into functional MLL1/2 complexes at the *DLX3* gene [18]. Since FLII is needed for SENP3 recruitment to the *DLX3* gene, we reasoned that loss of FLII would translate into an altered sumoylation status of RbBP5. To test this possibility, we introduced His-SUMO2 together with Flag-RbBP5 in HeLa cell and performed anti-Flag immunoprecipitation followed by anti-SUMO2/3 immunoblotting. Cells were either mock depleted or depleted from FLII by siRNA. Importantly, in anti-FLAG IPs, RbBP5–SUMO2 conjugates where enriched upon depletion of FLII (Fig. 5a) indeed suggesting that FLII is needed to channel SENP3 activity to RbBP5.

Since loss of FLII phenocopied the loss of SENP3 with respect to DLX3 expression, we asked whether FLII is also functionally connected to osteogenic differentiation. Primary human dental follicle stem cells (DFCs) were employed as an experimental system for the investigation of FLII function in this process. In the absence of SENP3, osteogenic differentiation is prevented due to improper induction of the DLX3, which is needed for the activation of downstream targets, such as RUNX2 and alkaline phosphatase (ALP). To investigate whether FLII is also involved in this pathway, we depleted endogenous FLII from DFCs and followed the expression of DLX3 upon induction of differentiation. In this experimental setup, loss of FLII resulted in a threefold to fourfold reduction of DLX3 mRNA and protein levels when compared to control cells (Fig. 5b–d). Accordingly, the downstream targets of DLX3, RUNX2 and ALP were also down-regulated (Additional file 1: Fig. S8a). Moreover, a significant decrease in ALP activity (Fig. 5e), which is indicative of defective osteogenic differentiation, was observed. Proper osteogenic differentiation of DFCs is characterized by intracellular deposition of calcium, which can be monitored by alizarin staining [54]. Importantly, FLII depletion caused drastically reduced alizarin staining indicating an impaired differentiation process (Fig. 5f and Additional file 1: Fig. S8b). Taken together, these findings establish a critical role of FLII in the control of osteogenic differentiation in human mesenchymal stem cells.

## Discussion

Our previous study unraveled a pathway, in which SENP3-mediated desumoylation of the RbBP5 subunit triggers the assembly of a functional MLL1/2 complex by recruitment of the Ash2L and menin subunits. Here, we define FLII as a central regulator of this pathway and provide evidence that FLII determines the chromatin recruitment of SENP3. FLII association with SENP3 and MLL1/2 complex is crucial for *HOX* gene expression. Mechanistically, we could delineate that FLII action triggers molecular events necessary for MLL1/2 complex association and subsequent epigenetic modifications permissive for *HOX* gene transcription required for human osteogenic differentiation.

FLII harbors an N-terminal leucine-rich repeat domain and two gelsolin-related domains, each of which is comprised of three repeats of around 150 amino acids [55]. Gelsolin domains define a family of actin-remodeling proteins, and accordingly FLII has been characterized as a regulator of cytoskeletal organization and actin dynamics. In addition to its role in actin remodeling, a transcriptional co-activator function in nuclear receptor signaling has been assigned to FLII. FLII interacts with estrogen receptor α (ERα) and the co-activators GRIP1 and CARM1 [50]. Moreover, FLII interacts with BAF53, a component of SWI/SNF chromatin remodeler complexes, and recruits SWI/SNF complex to its target genes [55]. Thus, FLII facilitates chromatin accessibility on ERα target genes, which was proposed to result in a proliferative advantage to breast cancer cells [56]. Our data are consistent with the dual roles for FLII as a transcriptional co-activator in the nucleus and a cytoplasmic modulator of the cytoskeleton. However, our work expands the regulatory function of FLII on transcriptional processes. We provide evidence that FLII is physically and functionally connected to MLL1/2 histone methyltransferase complexes and controls the recruitment of the desumoylating enzyme SENP3 to a subset of MLL1-/2-regulated *HOX* genes. Our interactome studies identified FLII as a very robust direct binding partner of SENP3. Binding is mediated predominantly through the GelA domain. Importantly, in the absence of FLII, SENP3 is not properly recruited to our model homeobox gene *DLX3*. As a consequence of reduced SENP3 occupancy, the MLL complex is not properly assembled, H3K4 trimethylation is impaired and expression of *DLX3* is down-regulated. Intriguingly, there is a striking overlap of *HOX* genes co-regulated by both FLII and SENP3. The expression of *HOXA9, HOXB3, MEIS1, MEOX1* and *DLX3* is all affected by depletion of either FLII or SENP3, while *HOXC8* expression remains unaltered in the absence of either SENP3 or FLII. Together with the fact that co-depletion of FLII and SENP3 is epistatic in controlling DLX3 expression, this strongly supports the idea that FLII acts on *HOX* gene expression through recruitment of SENP3. One possible scenario is that FLII, SENP3 and RbBP5 form a complex, in which FLII acts as a scaffold or adaptor that positions SENP3 in proximity to its substrate RbBP5. This allows desumoylation of RbBP5, which is a prerequisite for proper assembly of the MLL1/2 complexes at chromatin. Altogether, these data indicate that FLII contributes to SENP3 target selection by determining its recruitment to specific chromatin locations. How FLII acquires selectivity for addressing SENP3 to only a subset of MLL1/2 targets remains to be determined, but generally our data further strengthen the concept that substrate specificity of SENPs is dictated by spatial control. SENP3 stands as a paradigm for this concept as its activity is concentrated in the nucleolus, where it functions in ribosome maturation in complex with PELP1–TEX10–WDR18, and at transcriptionally active chromatin sites in conjunction with SET1/MLL methyltransferase complexes. Our data indicate that chromatin association of SENP3 requires FLII, while its nucleolar sequestration is mainly determined by the nucleolar scaffold protein nucleophosmin (NPM1) [11]. Noteworthy, FLII is largely excluded from the nucleolus making it unlikely that it is involved in the nucleolar functions of SENP3.

Our data also unravel an important and so far unprecedented implication of FLII in cell differentiation. In dental follicle progenitor cells, which serve as a model system of osteogenic differentiation, FLII depletion recapitulates the loss of SENP3 and impairs expression of the osteogenic master regulator DLX3. This in turn affects the expression of osteogenic regulators, such as RUNX2 and alkaline phosphatase, ultimately preventing proper osteogenesis. So far, FLII has been mainly linked to ERα-regulated transcriptional processes. Interestingly, however, at least on some *HOX* genes a cross talk between ERα activation and MLL-dependent H3K4 trimethylation has been reported [57–60]. Based on these observations, it is tempting to speculate that the FLII–SENP3 axis integrates multiple signaling pathways in cellular differentiation processes. Consistent with this idea, it has recently been shown that ERα regulates DLX3-mediated osteoblast differentiation [61]. Generally, our work expands the emerging concept of actin-binding proteins functioning in transcriptional processes. One paradigm for this concept is Wave1, which has a cytoplasmic function in actin reorganization as a downstream target of RAC. Intriguingly, however, reminiscent to our findings on FLII, nuclear Wave1, associates with SET proteins and modulates their activities on *HOX* genes [62]. Understanding how the nuclear and cytosolic functions of actin-binding proteins are coordinated will be one key question for future research.

## Conclusions

In summary, our work defines an unrecognized molecular mechanism that connects the actin-remodeling protein FLII to SENP3-sensitive *HOX* gene regulation. By recruiting SENP3 to chromatin at a subset of *HOX* genes, FLII coordinates MLL complex assembly and recruitment of active RNA polymerase II. FLII therefore functions as a critical specificity factor in the control of SENP3-mediated gene expression programs.

## Methods

### Cell culture/SILAC and transfection

HeLa cells were grown using standard conditions in Dulbecco’s modified Eagle’s medium (DMEM) supplemented with 10% fetal bovine serum and standard antibiotics. For SILAC labeling, cells were cultured in DMEMs (Thermo Scientific, product no. 89985) supplemented with 10% dialyzed serum (Invitrogen), antibiotics and amino acid isotope (R_0_K_0_ or R_6_K_4_, Cambridge Isotope Laboratories). Cells were grown for five passages to ensure the incorporation of labeled amino acids [63]. After five passages, incorporation of labeled amino acids to more than 95% was checked. siRNA-mediated knockdown experiments in HeLa cells were performed using Oligofectamine (Invitrogen) according to the manufacturer’s instructions.

### Mass spectrometry and data processing

Overnight in-gel trypsin (12.5 ng/μl) digested peptide mixture was desalted and purified by C-18 StageTip method. An Easy-nLCII liquid chromatography—coupled to a LTQ Orbitrap Elite mass spectrometer (Thermo Scientific)—was used for peptide elution and mass spec analysis. Desalted peptide mixtures were eluted with a 5–33% gradient HPLC solvent B (80% acetonitrile in 0.5% acetic acid). A mass range of *m*/*z* 150–2000 with a resolution of 120,000 was set to acquire full MS scan. Twenty most intense ions (Top20 method) were sequentially selected for CID fragmentation process. Data-dependent scanning mode with 90 s of dynamic inclusion was set for MS/MS scan. Data analysis was performed using MaxQuant software [64] version 1.3.0.5 with supported by Mascot as the database search engine for peptide identification. False discovery rate (FDR) was kept at 1% for data filtering, and two missed cleavages were allowed. Initial mass tolerance was set to 7 ppm and 0.5 Da for the fragment ion level. Cysteine carbamidomethylation was set as fixed modification, whereas protein N-terminal acetylation and oxidation of methionine were defined as variable modifications. Information about peptide MS/MS spectra was extracted from MaxQuant viewer. Cytoscape version 3.3.0 was used to generate protein network.

### Immunoprecipitation and chromatin immunoprecipitation

Immunoprecipitations were performed as described [18]. For endogenous SENP3 IP-MS, 80 millions SILAC-labeled cells were used. Cells were lysed in buffer containing 50 mM HEPES pH 7.5, 150 mM NaCl, 2 mM EDTA, 0.5% TritonX-100 with freshly added protease and phosphatase inhibitors (Complete/PhosphoSTOP, Roche). SENP3 IP (for 2 h) was performed with the lysate originated from R_6_K_4_, labeled cells and equal amount of R_0_K_0_ for control rabbit IgG IP. Immune complexes were captured by proteinG dynabeads (Invitrogen) for 1 h. After washing, immunoprecipitates were eluted by boiling the beads with SDS-PAGE loading buffer. Before loading in a SDS-PAGE gradient (4–20%, Biorad) gel, SENP3 IP and control IP were mixed into 1:1 ratio. ChIP assays were performed according to the published method from [65]. After real-time PCR of ChIPed DNA, data calculation was performed by percentage of input method. Two percentage starting chromatin was used as input. The diluted input was adjusted to 100% by subtracting raw diluted input Cp values with 5.64. If the starting input fraction is 2%, then a dilution factor of 50 is 5.64 cycles (i.e., log2 of 50 is 5.64). Finally, the percentage of input was derived from the following formula, i.e., 100 × 2^(Cp of adjusted input − Cp (ChIP replicates)). The average of at least three technical replicate was considered for a particular experimental setup. Unperturbed condition (such as control siRNA) was set as 100. Changes in chromatin occupancy were then calculated relative to 100. Data were presented as an average of three biological replicates (unless mentioned otherwise in the corresponding legend) with ± SEM. Details of western blot and ChIP antibodies are described in Supplemental information.

### In vitro transcription/translation and GST pull-down assay

One microgram of pCI vector encoding the respective proteins was translated with the TNT T7 quick-coupled transcription/translation system (catalog no. L1170) from Promega. GST-fused FLII domains were purification, and GST pull-downs were performed as described previously [66].

### Osteogenic differentiation of DFCs and measurement of alkaline phosphatase activity

Isolation, differentiation of DFC, siRNA-mediated protein knockdown and alkaline phosphatase assays were performed as described [18, 24, 54, 67, 68].

For the preparation of human dental follicle cells (DFCs), impacted human third molars were surgically removed and collected from patients with informed consent. DFCs were isolated as described [18, 54]. Differentiation of DFCs, siRNA-mediated protein knockdown and alkaline phosphatase assays were performed according to Nayak et al. [18].

### Alizarin red staining

Long-term cultures (after 5 weeks) were washed in PBS and fixed with 4% formaldehyde/0.1 M PBS. Calcium deposits were detected by treatment with a 2% alizarin red S solution (Morphisto). For quantitative measurement, the alizarin red staining was solved in a 10% cetylpyridinium chloride monohydrate solution (PBS) for 30 min. The optical density of samples was measured in a plate reader at 540 nm.

### Quantitative reverse transcription polymerase chain reaction (RT-qPCR)

For total RNA isolation, high-pure RNA isolation kit (Roche) was used. Five hundred nanograms of that was used for cDNA synthesis (Transcriptor First strand cDNA synthesis kit, Roche) by using oligo dT primer. RT-qPCR was performed with Fast Start DNA Master SYBR^®^ Green I kit (Roche) and the Light Cycler PCR System (LightCycler 480 II, Roche). For normalization, GAPDH gene expression was used as a control gene expression marker. Quantification of mRNA expression was performed with the standard curve method.

### SENP3 catalytic activity assay

About 10 million HeLa cells (Control siRNA in parallel with FLII siRNA for 72 h) were lysed in 1 ml SEM buffer (0.25 M sucrose, 20 mM MOPS–KOH and 1 mM EDTA–NaOH, pH 8) with or without 20 mM *N*-ethylmaleimide (NEM). Protease inhibitor cocktail and 1 mM DTT were added freshly. After brief sonication (3 strokes for 5 s at 40% amplitude), protein concentration was measured by Bradford assay. Two hundred micrograms of extract was diluted in activity assay buffer (50 mM Tris–HCl pH 7.5, 0.1 mg/ml BSA and 10 mM DTT) and mixed with 100 ng SUMO-2 vinyl sulfone derivative (HA-SUMO2-VS; Boston Biochem) compound. The reaction was kept at room temperature for 10 min. To stop the reaction, sample buffer was added followed by heating at 95° for 5 min.

### Immunofluorescence

HeLa cells were fixed in methanol (MeOH) at −20 °C for 5 min, permeabilized with 0.5% Triton X-100 and processed using standard protocols. Images were acquired by Leica TCS SP8 confocal microscope. An anti-SENP3 (clone D20A10 cat no. 5591, Cell signaling) and anti-FL II (SC-21716, Santa Cruz Biotechnology Inc.) were used as primary antibodies for detecting the respective proteins. The following secondary antibodies were used: Alexa Fluor 488–donkey anti-rabbit (Invitrogen), Cy3–donkey anti-mouse (Invitrogen).

## Additional files

**Additional file 1: Fig S1.** Proteome map of SENP3 derived from SILAC-based mass spectrometry. (a) Schematic representation of SENP3 proteomics. Equal no. of HeLa cells (as mentioned in “Methods”) either unlabeled or metabolically labeled with amino acid isotope (R6K4) was used for IP. Control IP and SENP3 IP were mixed in a 1:1 ratio and loaded in a SDS-PAGE. The whole lane was cut into several small pieces and processed for mass spec (as described in Materials and Methods section). (b) One representative western blot shows the enrichment of endogenous SENP3 in IP lane that was used for MS analysis. (c) The cytoscape map of SENP3 interactome was accomplished after filtering the whole protein group file (generated from MaxQuant analysis) through 4 tier of following selection criteria—(i) normalized H/L SILAC ratio cutoff was set as 2; that is proteins with minimum twofold enrichment compare to IgG control were considered. (ii) PEP score cutoff was set as (0.0001). PEP score is like *p* value that represents statistical significance of an observed peptide as a true one. Therefore, smaller PEP score is significant. (iii) Minimum three peptides were considered for any proteins and (iv) reproducibility in both the independent experiments. (d) Cytoscape network of SENP3 interactome obtained from two independent SILAC-MS assays. Details of the generation of the cytoscape map are described in appendix figure S1c. (e) A representative MS/MS spectrum of FLII peptide that was generated by MaxQuant Viewer program. **Fig S2.** SENP3 protein network. (a) The filtered protein candidates (27) generated from SENP3 proteomics were entered into STRING database as input to check for clustering coefficient. Red arrow indicates the bait, SENP3. (b) Information about FLII protein–protein interaction was extracted from STRING database and combined together with SENP3 network from our experiment. **Fig S3.** FLII–SENP3 interaction is mostly in the nucleus. (a) Related to Fig. 1. HeLa cells were transfected with Flag-SENP3. Two days after transfection, Flag-agarose bead pull down was performed to check the presence of FLII in the western blot. (b) Subcellular fractionation of HeLa cells was performed according to the user protocol (subcellular protein fractionation kit, ThermoFisher Scientific, catalog no. 78840). Endogenous FLII was immunoprecipitated from cytosolic and nuclear fraction. SDS-PAGE of the immunoprecipitate was performed and probed for indicated antibodies. (c) Subcellular localization of endogenous SENP3 and FLII was studied by immunofluorescence using primary antibodies detecting the respective proteins. **Fig S4.** FLII interaction with RbBP5. (a) Related to Fig. 3. Endogenous FLII was immunoprecipitated from HeLa cells, and blot was probed against indicated antibodies. (b) Related to Fig. 3. Endogenous SENP3 was immunoprecipitated from HeLa cells, and blot was checked for indicated antibodies. (c) Related to Fig. 3. Endogenous WDR5 was immunoprecipitated, and blot was probed against indicated antibodies. (d) Same as Fig. 1d except RbBP5 construct was used for in vitro transcription/translation. (e) Same as additional file 1, Fig S3a, except Flag-tagged FLII constructs were used to check the interaction of various FLII domains (as indicated in the figure) with RbBP5. **Fig S5.** SENP3 is not involved in ERα and FLII does not influence SENP3 catalytic activity. (a) Post-transfection (control, SENP3 and FLII siRNA), MCF7 cells were cultured for 3 days in phenol red-free DMEM medium supplemented with 5% charcoal-dextran-stripped fetal bovine serum. Cells were then treated overnight with 100 nM estradiol (E2) before RNA extraction. Data represent the average of triplicates from two biological experiments ± SEM. (b) 72 h after siRNA treatment directed against FLII, cell lysate was prepared in the presence or absence of NEM in the lysis buffer. Equal amount of protein (200μg) of protein from control and FLII siRNA cell lysate was mixed with SUMO2-VS [69] substrate at room temperature for 10 min. The SDS-PAGE was probed by using SENP3 and a loading control tubulin antibody. The asterisk mark represents slow-migrating catalytic active SENP3 form appeared as a result of conjugation between the substrate and SENP3. Right panel shows FLII knockdown efficiency. (c) Same as in (a), except anti-HA antibody (that detects SUMO2-VS substrate) was used. **Fig S6.** FLII influences MLL1/2 complex assembly on *DLX3* gene. (a) Same as Fig. 4b except 5μg of flag-FLII plasmid was transfected to HeLa cells. 48 h. post-transfection cells were fixed and processed for ChIP using rabbit flag antibody. Data represent average of at least two biological experiments performed in duplicate. (b) Related to Fig. 4B. HeLa cell lysate was probed with indicated antibodies to monitor the depletion of FLII and SENP3. (C) Same as Fig. 4e, but DLX3.2 primer was used in qPCR (n = 3, T test, *p < 0.05; **p < 0.01; ***p < 0.001, n.s. not significant). (d) Same as Fig. 4e, except anti-MLL1 and anti-MLL2 antibodies were used for ChIP. DLX3.1 primer was used for qPCR. (e) Same as in d, except DLX3.2 primer was used for qPCR. **Fig S7.** Expression of MLL1/2 complex subunits is unperturbed upon FLII depletion. (a, b) To check the effect of FLII knockdown on MLL1/2 complex subunit protein stability, cell lysates from control and FLII siRNA were probed in western blot for the indicated antibodies. (c) Same as Fig. 4e except *HOXC8* promoter primer was used (n = 3, n.s. not significant). (d, e) Related to Fig. 4f and g except *HOXC8* promoter primer was used. (f) HeLa cells were transfected with FLII siRNA for 72 h followed by endogenous SENP3 and RbBP5 immunoprecipitation. Blot was probed with indicated antibodies. (g) Same as (f) but SENP3 siRNA was performed and RbBP5 antibody was used for immunoprecipitation. **Fig S8.** FLII is required for osteogenic differentiation. (a) Expression of RUNX2 and ALP mRNA was determined by RT-qPCR in control or DFCs depleted from FLII. Values (normalized for GAPDH expression) represent the mean of three experiments ± SEM. (b) The status of mineral deposition in DFC was measured by alizarin stain. Cells were transfected with two different FLII siRNA and grown in ODM culture for 5 weeks.

**Additional file 2: Table S1.** Table shows the detail of proteins that passed the filtering criteria of Fig S1.

**Additional file 3.** Supplementary materials and methods.

## Abbreviations

Ash2L: absent, small or homeotic 2-like
MLL: mixed lineage leukemia
RbBP5: retinoblastoma-binding protein 5
SUMO: small ubiquitin-like modifier
SENP3: sentrin-specific protease 3
pol II: RNA polymerase II
DFC: dental follicle stem cells
FLII: flightless-I homolog
