# Supplementary figures and images for: Flightless-I governs cell fate by recruiting the SUMO isopeptidase SENP3 to distinct HOX genes
Source: Epigenetics Chromatin. 2017 Mar 23;10:15. doi: 10.1186/s13072-017-0122-8 (PMC5364561; doi:10.1186/s13072-017-0122-8)

Figure S1

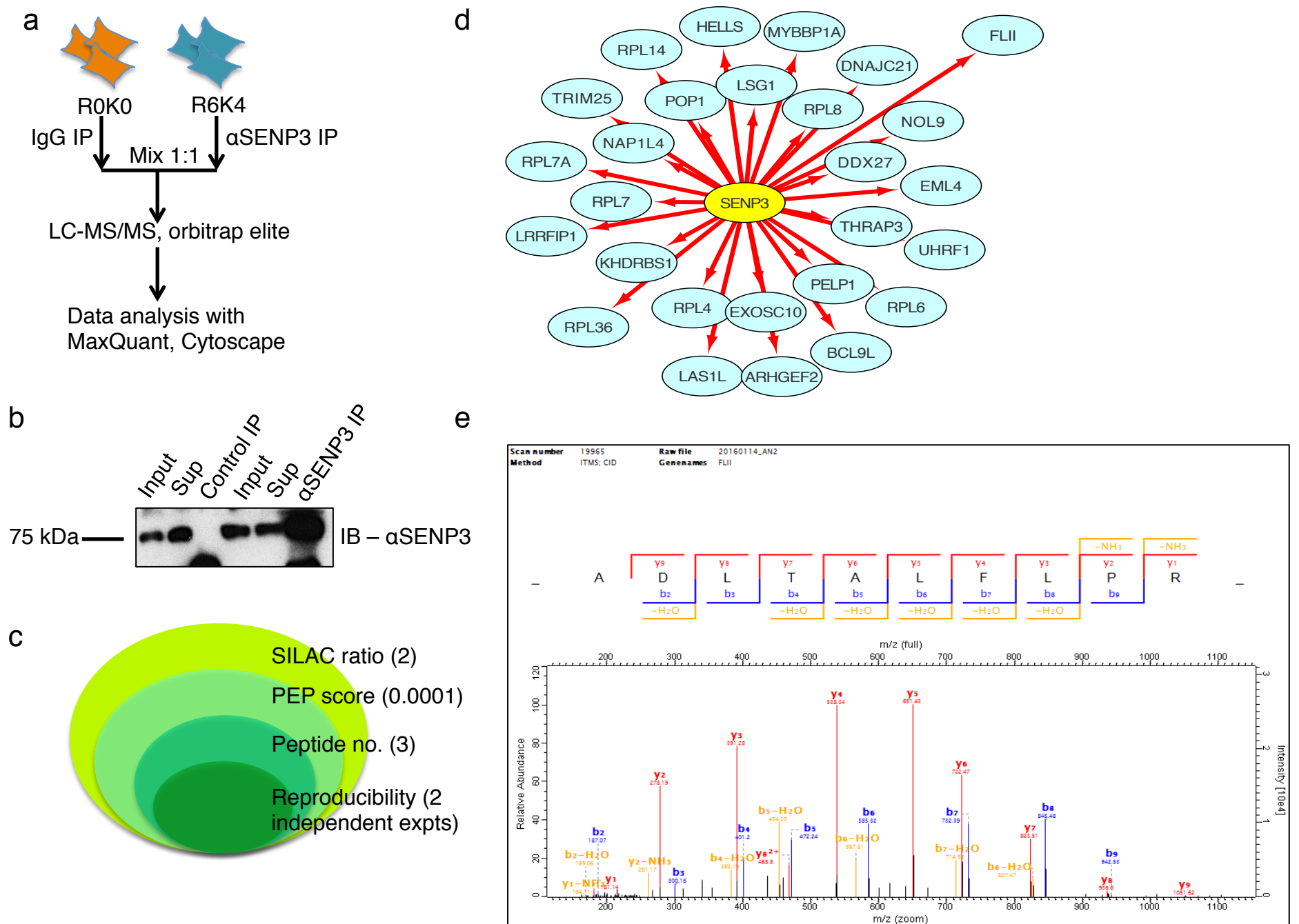

a

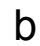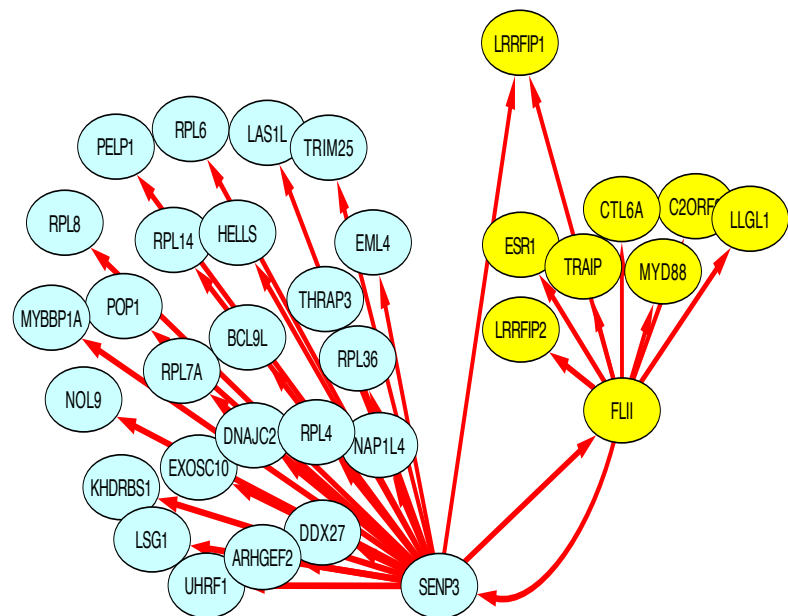

Figure S3

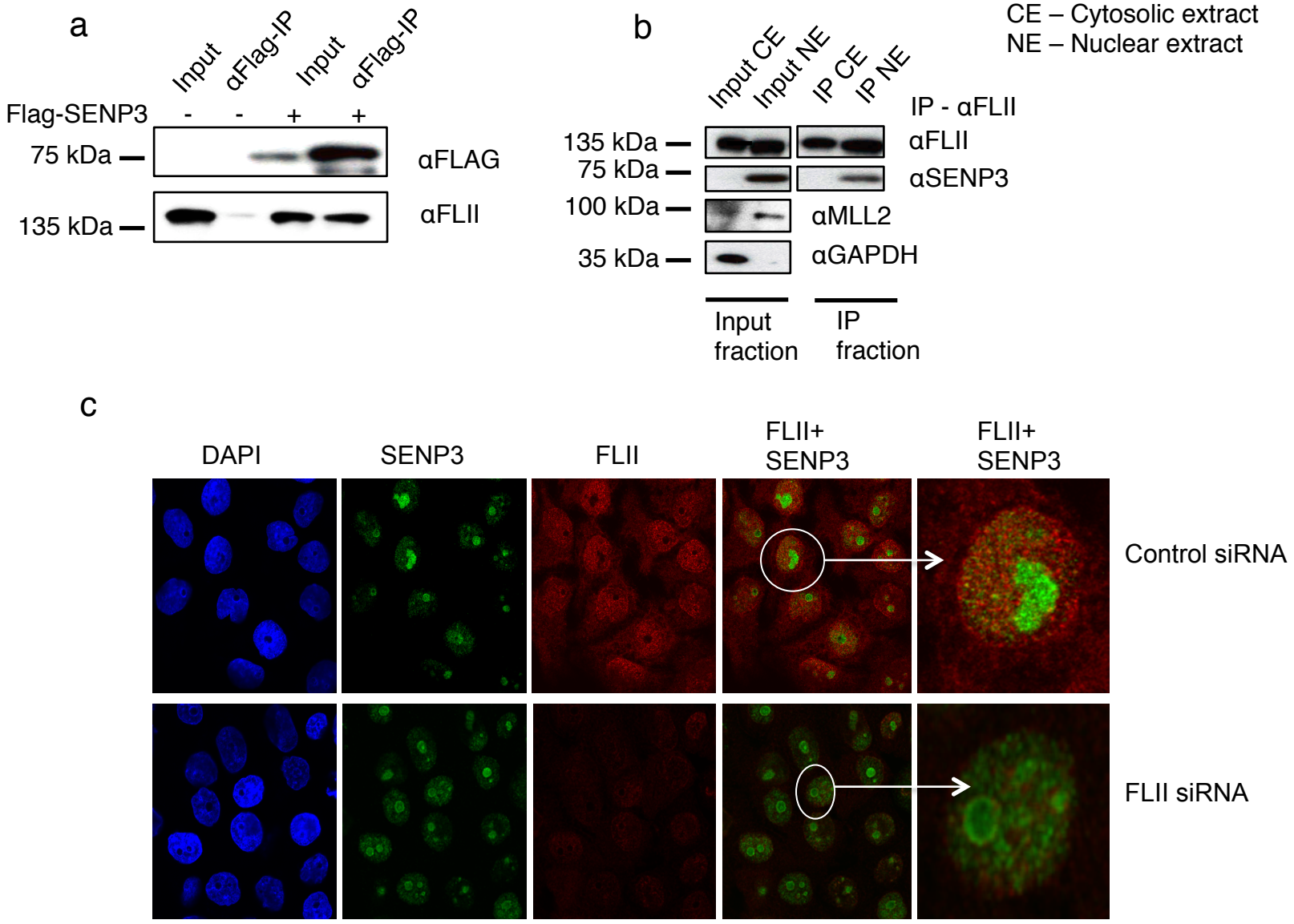

Figure S4

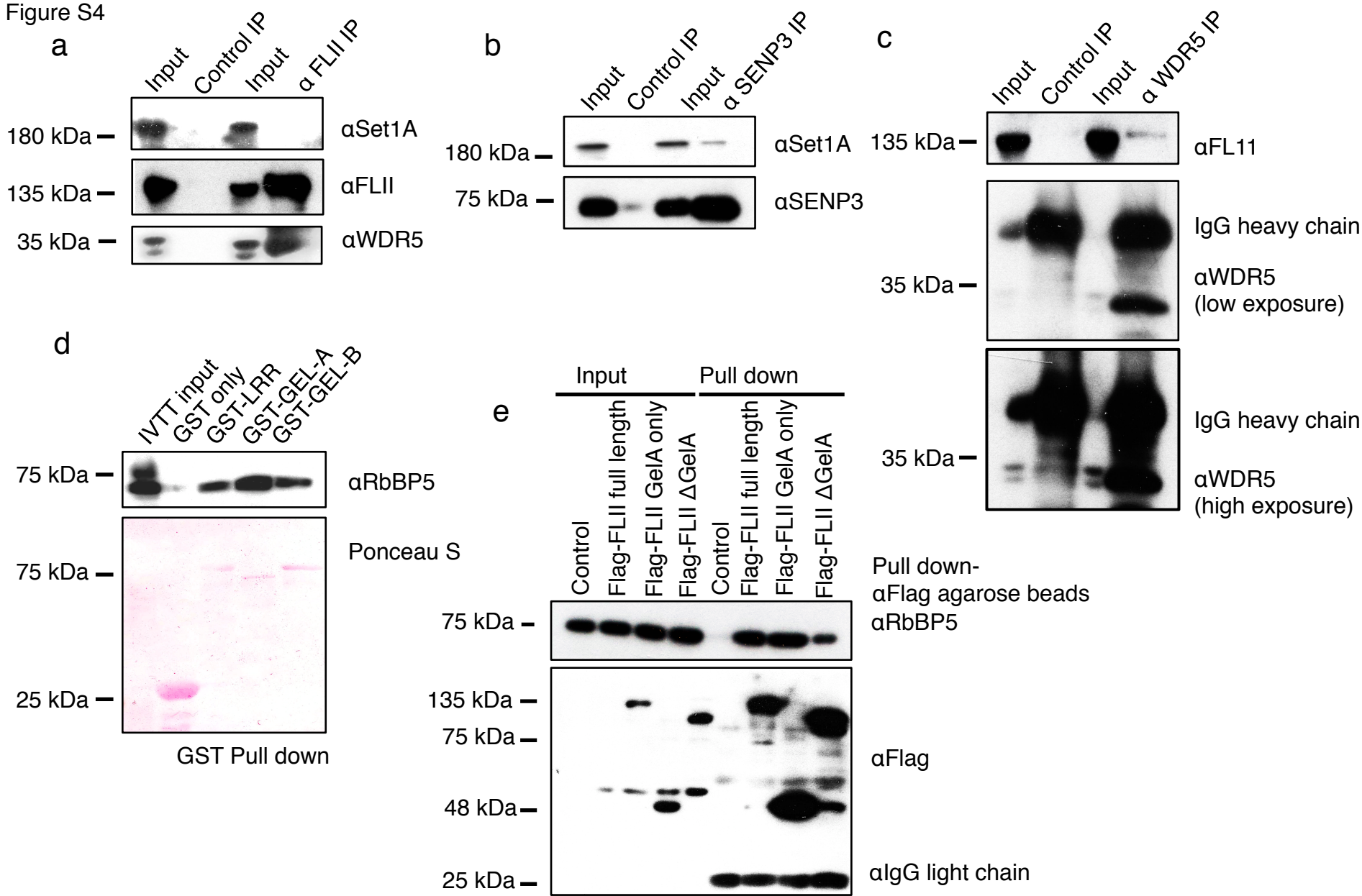

Figure S5

a

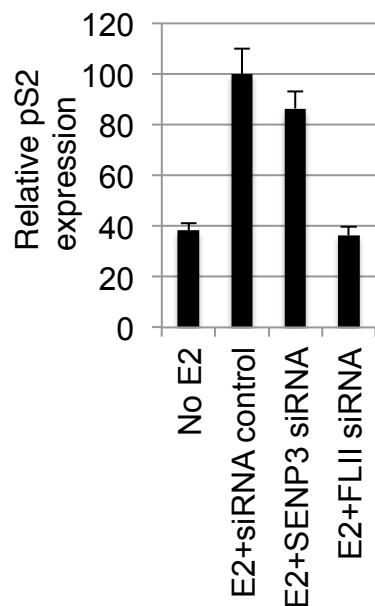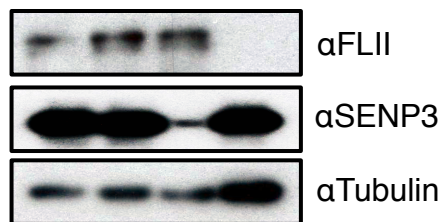

b

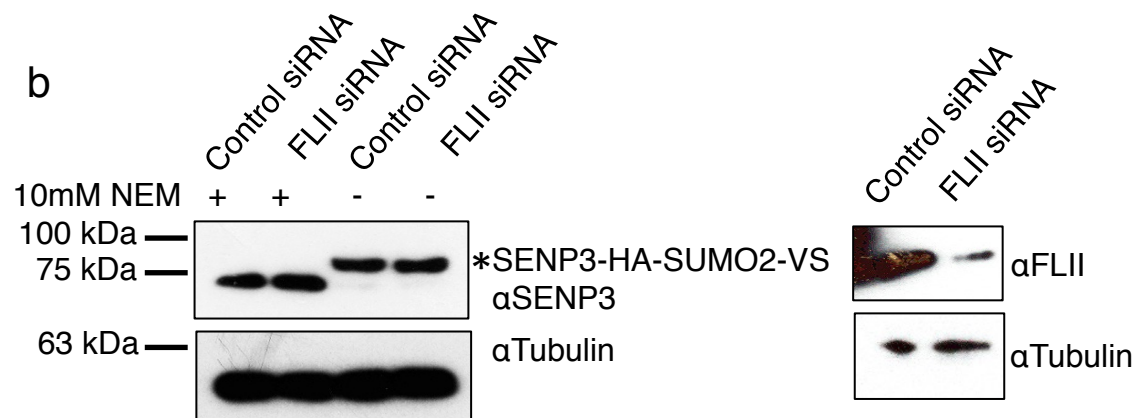

c

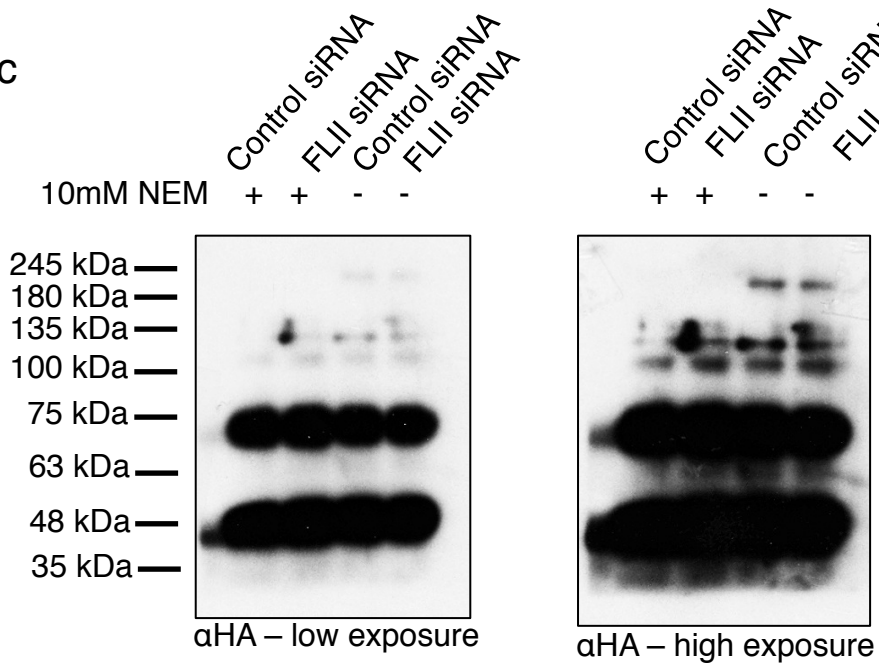

Figure S6

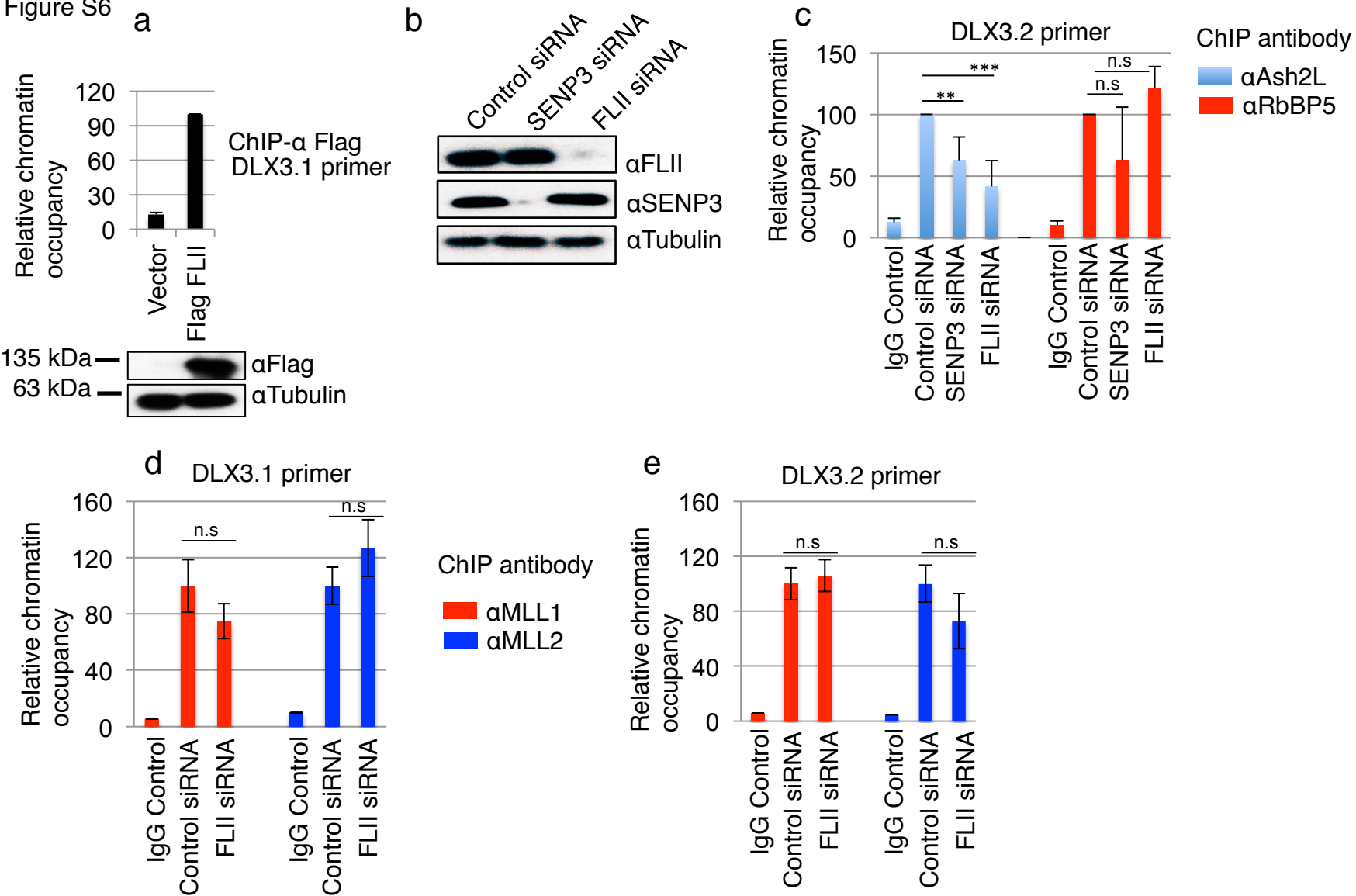

Figure S7

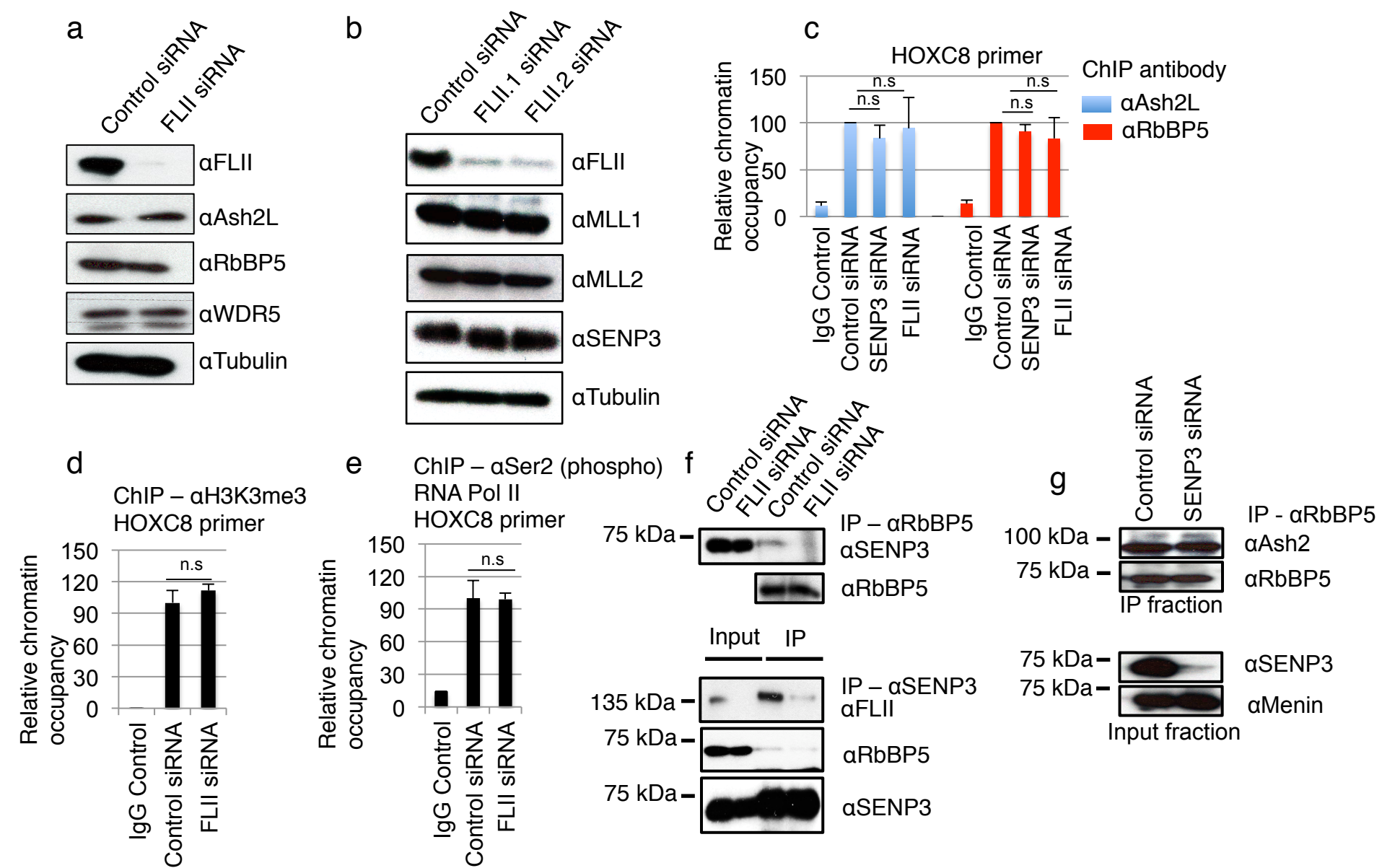

Figure S8

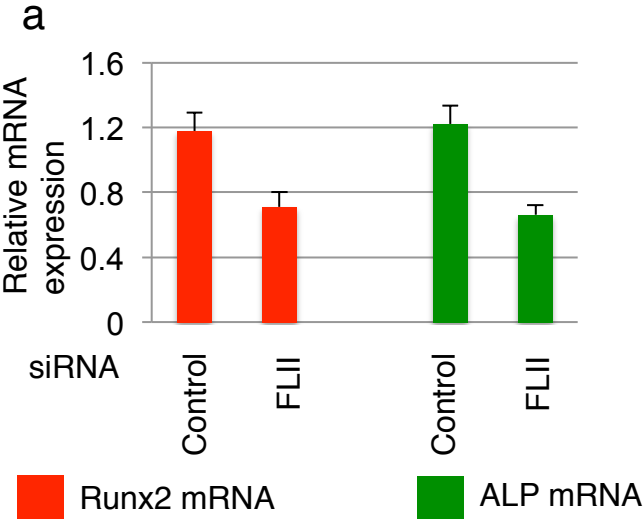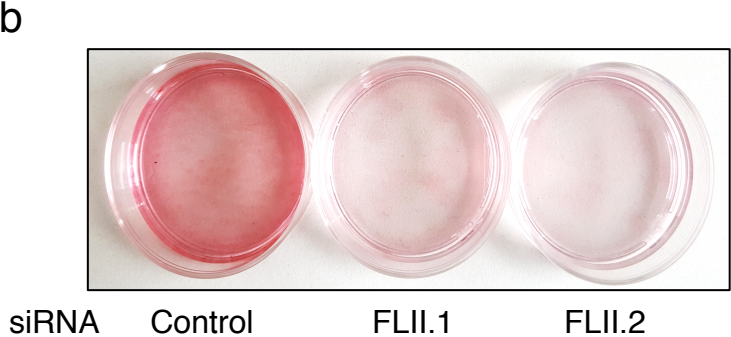

Supplement: Supplementary file 1 — Additional file 1: Fig S1. Proteome map of SENP3 derived from SILAC-based mass spectrometry. (a) Schematic representation of SENP3 proteomics. Equal no. of HeLa cells (as mentioned in “Methods”) either unlabeled or metabolically labeled with amino acid isotope (R6K4) was used for IP. Control IP and SENP3 IP were mixed in a 1:1 ratio and loaded in a SDS-PAGE. The whole lane was cut into several small pieces and processed for mass spec (as described in Materials and Methods section). (b) One representative western blot shows the enrichment of endogenous SENP3 in IP lane that was used for MS analysis. (c) The cytoscape map of SENP3 interactome was accomplished after filtering the whole protein group file (generated from MaxQuant analysis) through 4 tier of following selection criteria—(i) normalized H/L SILAC ratio cutoff was set as 2; that is proteins with minimum twofold enrichment compare to IgG control were considered. (ii) PEP score cutoff was set as (0.0001). PEP score is like p value that represents statistical significance of an observed peptide as a true one. Therefore, smaller PEP score is significant. (iii) Minimum three peptides were considered for any proteins and (iv) reproducibility in both the independent experiments. (d) Cytoscape network of SENP3 interactome obtained from two independent SILAC-MS assays. Details of the generation of the cytoscape map are described in appendix figure S1c. (e) A representative MS/MS spectrum of FLII peptide that was generated by MaxQuant Viewer program. Fig S2. SENP3 protein network. (a) The filtered protein candidates (27) generated from SENP3 proteomics were entered into STRING database as input to check for clustering coefficient. Red arrow indicates the bait, SENP3. (b) Information about FLII protein–protein interaction was extracted from STRING database and combined together with SENP3 network from our experiment. Fig S3. FLII–SENP3 interaction is mostly in the nucleus. (a) Related to Fig. 1. HeLa cells were transf [file 13072_2017_122_MOESM1_ESM.pdf]
