# Supplementary material for: Flightless-I governs cell fate by recruiting the SUMO isopeptidase SENP3 to distinct HOX genes
Source: Epigenetics Chromatin. 2017 Mar 23;10:15. doi: 10.1186/s13072-017-0122-8 (PMC5364561; doi:10.1186/s13072-017-0122-8)
Supplement: Supplementary file 2 — Additional file 2: Table S1. Table shows the detail of proteins that passed the filtering criteria of Fig S1. [file 13072_2017_122_MOESM2_ESM.pdf]

Table S1

Experiment 1 Experiment 2

Gene Name	No. of peptide	Normalized SILAC ratio (H/L)	PEP score		No. of peptide	Normalized SILAC ration (H/L)	PEP score	Mol. Wt.
SENP3	23	15,48	2,91E-173		21	3,012	1,37E-189	65,009
LAS1L	13	16,41	8,62E-128		16	3,158	2,80E-98	83,064
ARHGEF2	15	13,56	4,64E-69		13	2,304	6,39E-64	111,49
RPL7	14	10,335	7,56E-77		12	2,609	9,91E-116	29,225
RPL8	7	9,42	2,01E-28		5	2,086	2,10E-29	28,024
RPL14	6	9,226	5,91E-29		3	2,391	5,91E-12	23,432
DNAJC21	4	9,13	3,85E-14		3	2,383	6,41E-11	62,027
RPL36	3	8,174	1,09E-09		3	2,173	6,54E-23	12,254
KHDRBS1	3	7,75	5,11E-12		3	2,091	2,30E-09	48,227
RPL6	12	7,201	5,87E-86		10	2,602	7,51E-16	32,728
EML4	10	7,138	7,70E-23		9	2,169	1,66E-16	108,91
FLII	55	7,107	0		47	3,143	0	144,75
RPL7A	10	7,062	1,07E-43		7	2,293	1,23E-78	29,995
LRRFIP1	46	6,094	0		40	3,391	0	89,252
TRIM25	16	5,5	3,94E-227		9	2,645	3,65E-49	70,973
NAP1L4	6	5,175	6,05E-68		4	3,015	1,66E-82	44,078
POP1	11	4,953	6,32E-33		8	2,298	1,41E-29	114,71
THRAP3	7	4,629	9,60E-22		13	2,601	8,04E-38	108,66
BCL9L	21	4,139	4,76E-109		13	2,593	2,58E-21	157,13
LSG1	10	3,502	1,97E-49		6	2,178	2,58E-21	75,225
RPL4	13	3,471	6,85E-43		6	2,248	1,07E-16	33,3
PELP1	6	3,148	1,32E-16		3	2,341	3,40E-04	135,51
NOL9	4	3,142	3,38E-11		3	4,324	3,40E-04	79,322
MYBBP1A	21	2,913	9,87E-55		18	2,059	1,61E-47	148,85
EXOSC10	8	2,619	7,71E-35		9	2,038	9,70E-14	109
UHRF1	6	2,219	9,22E-39		5	2,139	3,54E-17	89,813
DDX27	12	2,203	9,38E-48		8	2,029	2,30E-44	89,834
HELLS	5	2,182	9,32E-62		3	2	2,26E-25	97,073
