# Supplementary material for: Flightless-I governs cell fate by recruiting the SUMO isopeptidase SENP3 to distinct HOX genes
Source: Epigenetics Chromatin. 2017 Mar 23;10:15. doi: 10.1186/s13072-017-0122-8 (PMC5364561; doi:10.1186/s13072-017-0122-8)
Supplement: Supplementary file 3 — Additional file 3. Supplementary materials and methods. [file 13072_2017_122_MOESM3_ESM.pdf]

## Supplemental Experimental Procedures

### Antibodies used for western blot, immunoprecipitation and ChIP

Details of antibodies used for western blot, immunoprecipitation and ChIP are as follows: anti-Ash2L II (A300-489A, Bethyl lab), anti-Menin (cat no. - A300-105A, Bethyl lab), anti-MLL1 (cat no. - A300-086A, A300-374A, Bethyl lab), anti-MLL2/TRX2 (cat no. - A300-113A, Bethyl lab), MLL2/ALR (ABIN731573), anti-RbBP5 (cat no. - A300-109A, Bethyl lab), anti-WDR5 (cat no. - A302-429A Bethyl lab), anti-SET1 (cat no. - A300-086A, Bethyl lab), anti-LAS1L (AV34629, Sigma-Aldrich), anti-FL II (SC-21716, Santa Cruz Biotechnology Inc), anti-LRRFIP1(A303-078A, Bethyl lab), anti-PELP1(A300-180A, Bethyl lab), anti-SENP3 (clone D20A10 cat no. - 5591, Cell signaling), anti-DLX3 (Abcam), anti-Flag (F3165, Sigma-Aldrich) anti-SUMO2/3 (clone 1E7, MBL), anti-RUNX2 (Santa Cruz Biotechnology Inc), anti-RNA Pol II (phospho ser 2) (cat no. – Ab5095, Abcam), anti-RGS His (cat no. – 34610, Qiagen), anti-$\alpha$tubulin (cat no. – CP06 Calbiochem), anti-H3K4 me3 (cat no. – ab8580, Abcam).

### siRNA sequences and PCR primers

The following siRNA oligos were used in knockdown assays:

Control- CGUACGCGGAAUACUUCGA dTdT

SENP3- ACGUGGACAUCUUCAAUAA dTdT

FL II.1 – GCUGGAACACUUGUCUGUG dTdT

FL II.2 – CAACCUGACCACGCUUCA dTdT

The following primer pairs were used for RT-qPCR to amplify cDNA:

GAPDH forward 5’-GGTATCGTGGAAGGACTCATGAC-3’

GAPDH reverse 5’-ATGCCAGTGAGCTTC CCGTTCAG-3’

SENP3 forward 5’-CAAAGTCTCCTCTGGACCCTG -3’

SENP3 reverse 5’-TGCTGCACACATTGCTGATGAG -3’

DLX3 forward 5´-ATCGCAAGCTCAGCAGCATC-3’

DLX3 reverse 5´-TAGCCCAGGTCAGTGACAGAAG-3’

HOXC8 forward 5´-ACGTGGACTCGCTCATCTCT-3’

HOXC8 reverse 5´-CGAAGCTACAGGACGGAAAA-3’

HOXA9 forward 5´-TAAACCTGAACCGCTGTCGGCCAG -3’

HOXA9 reverse 5´-CCGCCGCTCTCATTCTCAGCATTG -3’

HOXB3 forward 5´- AGTTCCATTTTAACCGCTACC -3’

HOXB3 reverse 5´- TTCTTGTACTTCATGCGCC -3’

MEIS1 forward 5´- TGTGACAATTTCTGCCACC -3’

MEIS1 reverse 5´- CTGAACGAGTAGATGCCGT -3’

MEOX1 forward 5´- TGCCCATCATAACTACCTGAC -3’

MEOX1 reverse 5´- ACACGCTTCCACTTCATCC -3’

RUNX2 forward 5´- GTGCCTAGGCGCATTTCA-3’

RUNX2 reverse 5´- GCTCTTCTTACTGAGAGTGGAAGG-3’

ALP forward 5´- CAACCCTGGGGAGGAGAC-3’

ALP reverse 5´- GCATTGGTGTTGTACGTCTTG-3’

FLII forward 5´- CCTCCTACAGCTAGCAGGTTATCAAC-3’

FLII reverse 5´- GCATGTGCTGGATATATACCTGGCAG -3’

The following primer pairs were used for the ChIP qPCR to amplify DNA derived from chromatin:

GAPDH forward 5’- TACTAGCGGTTTTACGGGCG-3’

GAPDH reverse 5’- TCGAACAGGAGGAGCAGAGAGCGA-3’

DLX3.1 forward 5´- ATCAGCGCGTAGGAGCCTC-3’

DLX3.1 reverse 5- CGTCCCAAGCCACAATCAAAT-3’

DLX3.2 forward 5´- CGCCGTTCCAAGTTCAAGAAAC-3’

DLX3.2 reverse 5´- TGATGGTGGTGAGTTGCAGG-3’

HOXC8 forward 5´- TCTCGCCTGTCTTCATGTC-3’

HOXC8 reverse 5´- TCTCTCACTTAGCTCTTTCCTC-3’
